# Benefits of HIV‐1 transmission cluster surveillance: a French retrospective observational study of the molecular and epidemiological co‐evolution of recent circulating recombinant forms 94 and 132

**DOI:** 10.1002/jia2.26416

**Published:** 2025-01-28

**Authors:** Marc Wirden, Fabienne Tombette, Sidonie Lambert‐Niclot, Marie‐Laure Chaix, Stéphanie Marque‐Juillet, Magali Bouvier‐Alias, Benedicte Roquebert, Moise Machado, Veronique Avettand‐Fenoel, Pierre Gantner, Enagnon Kazali Alidjinou, Karl Stefic, Jean‐Christophe Plantier, Vincent Calvez, Diane Descamps, Anne‐Genevieve Marcelin, Benoit Visseaux, the ANRS‐MIE resistance study group

**Affiliations:** ^1^ Sorbonne Université, INSERM, Institut Pierre Louis d'Epidémiologie et de Santé Publique IPLESP, AP‐HP Hôpital Pitié Salpêtrière, Laboratoire de virologie Paris France; ^2^ Univ Rouen Normandie, UNICAEN, INSERM, DYNAMICURE UMR 1311, CHU de Rouen, Service de virologie Centre National de référence VIH Rouen France; ^3^ AP‐HP, Hôpital Saint‐Antoine Service de Virologie Paris France; ^4^ AP‐HP, Hôpital Saint‐Louis Service de Virologie, INSERM U944 Paris France; ^5^ Hôpital de Versailles Service de Virologie Le Chesnay France; ^6^ HU Henri Mondor Service de Virologie Créteil France; ^7^ Laboratoire CERBA Saint Ouen L'Aumône France; ^8^ Grand Hôpital de l'Est Francilien, Site Marne‐La‐Vallée Service des Maladies Infectieuses et Tropicales Jossigny France; ^9^ AP‐HP, Hôpital Necker Service de Virologie Paris France; ^10^ HU de Strasbourg Service de Virologie Strasbourg France; ^11^ CHU de Lille Service de Virologie Lille France; ^12^ CHU de Tours Service de Virologie Tours France; ^13^ AP‐HP, Hôpital Bichat Claude Bernard Service de Virologie, INSERM, IAME Paris France

**Keywords:** cluster of transmission, CRF132, CRF94, HIV prevention, HIV‐1, molecular surveillance

## Abstract

**Introduction:**

Molecular surveillance is an important tool for detecting chains of transmission and controlling the HIV epidemic. This can also improve our knowledge of molecular and epidemiological factors for the optimization of prevention. Our objective was to illustrate this by studying the molecular and epidemiological evolution of the cluster including the new circulating recombinant form (CRF) 94_cpx of HIV‐1, detected in 2017 and targeted by preventive actions in 2018.

**Methods:**

In June 2022, 32 HIV‐1 sequence databases from French laboratories were screened to identify all individuals who had acquired CRF94_cpx or a similar strain, whatever the date of diagnosis. Phylogenetic analyses were performed with the sequences identified, and biological parameters were collected at the time of diagnosis and after the start of treatment to analyse the evolution of the cluster. Full genomes were sequenced to characterize the new strains.

**Results:**

We analysed 98 HIV‐1 isolates: 63 were CRF94, three were unclassifiable, and the other 32 formed a new cluster containing a new recombinant, CRF132_94B, derived from CRF94 and a subtype B strain. At least 95% of the individuals in both the CRF94 and CRF132 clusters were men who have sex with men (MSM), most of whom had acquired HIV less than 12 months before diagnosis. The number of CRF94 diagnoses declined drastically after 2018, but CRF132 strains spread widely between 2020 and 2022, into a different area of Ile‐de‐France region and within a younger population nevertheless aware of pre‐exposure prophylaxis. Higher viraemia, lower CD4 cell counts and delayed treatment efficacy suggested that CRF94 was more virulent than CRF132, possibly due to the F subtype fragment of the *vif* gene.

**Conclusions:**

These findings highlight the role of the MSM transmission cluster in spreading HIV and new variants. They show also the benefits of cluster surveillance for improving the targeting of preventive interventions, detecting the emergence of new strains and enriching our knowledge on virulence mechanisms. However, these investigations require support with sufficient resources dedicated to a regional or national programme to be responsive and effective.

## 1 INTRODUCTION

In 2023, HIV was still implicated in 630,000 deaths worldwide and 39.9 million people were living with the virus [1]. The epidemic predominantly affects Africa, but people are still acquiring HIV in Western countries [2, 3]. The universal test‐and‐treat strategy recommended by the WHO, in line with the UNAIDS concept that “undetectable = untransmittable,” and the scaling up of pre‐exposure prophylaxis (PrEP) in individuals at risk are consensual strategies for decreasing the spread of HIV worldwide [4, 5, 6]. However, surveillance based on molecular epidemiology using sequencing data from genotypic resistance testing is also useful for identifying expanding clusters of HIV transmission and targeting preventive actions [7, 8, 9, 10, 11, 12, 13, 14]. Such analyses are not only valuable for prevention, but can also rapidly detect the emergence of more virulent strains, which may then become the subject of special surveillance [15, 16]. Furthermore, the sustained nature of the HIV epidemic is allowing new circulating recombinant forms (CRFs) to emerge, especially among men who have sex with men (MSM), as recently reported [17]. Recombinant forms are now the most prevalent HIV‐1 strains worldwide [17]. Genetic recombination can facilitate the selection of more virulent HIV strains. This study, reporting the molecular and epidemiological evolution of two CRFs recently identified in France, perfectly illustrates all these points and the value of transmission cluster surveillance.

In 2018, we reported the discovery of a new CRF of HIV‐1, CRF94_cpx, in a major transmission cluster of MSM in France [15]. This new strain contained genomic segments from HIV‐1 subtypes B, CRF02 and F2. The biological characteristics of this new strain, resulting in very high viral loads and low CD4 cell counts in persons at diagnosis, raised concerns about enhanced virulence. The first patients to acquire this strain were diagnosed in 2013, but the cluster spread rapidly in the Ile‐de‐France region (IDF) in 2016–2017, subsequently spreading into other French regions. After the first study in 2017, CRF94 acquisitions continued to be diagnosed, but increasing numbers of individuals were found to have acquired a closely related, but not quite identical strain. This led to suspicions that the original CRF94 strain might have undergone molecular evolution, with an expansion of the transmission cluster. The objectives of this study were to survey the epidemiological and molecular evolution of CRF94 from 2018 to 2022, via the network of laboratories of the *Agence Nationale de Recherche sur le SIDA et Maladies Infectieuses Emergente*s, (ANRS‐MIE) in France. We also assessed the impact of targeted preventive actions carried out in 2018, based on HIV screening days, the promotion of condom use, and the distribution of information about post‐ and pre‐exposure prophylaxis in the geographical area in which the CRF94 cluster occurred.

## 2 METHODS

### 2.1 Data collection

In accordance with French and European guidelines, all HIV isolates from newly HIV‐diagnosed persons were sequenced to check for the presence of transmitted resistant strains [18, 19]. The HIV sequences obtained were also used to determine the subtype or CRF with Basic Local Alignment Search Tools (BLAST), through comparisons of the sequence with a pool of reference HIV‐1 sequences. In June 2022, the 36 laboratories of the ANRS‐MIE network throughout France (see the Acknowledgements section) screened their databases to identify all individuals diagnosed with an HIV subtype CRF94 or another strain with a similar pattern of recombination (i.e. protease [PR] and integrase [INT] genes matching the reference sequences for CRF02_AG, and a reverse transcriptase [RT] gene matching the reference sequences for subtype B). These HIV‐1 nucleotide sequences were collected, whatever the date of HIV diagnosis but with the exclusion of those already obtained during the 2017 study. Epidemiological and clinical data, sex, age, HIV‐1 viral loads and CD4 cell counts were collected at diagnosis and after treatment initiation, for all new patients and for those with CRF94 already included in the previous study but for whom follow‐up was too short for analysis in 2017.

### 2.2 Phylogenetic and molecular analysis

We analysed the homology and clustering of newly identified viruses with all available previous CRF94_cpx strains, by performing phylogenetic analyses based on the protease/reverse transcriptase (1070 bp) and integrase (696 bp) genes. ClustalW in MEGA 11 software was used to align all sequences with the reference sequences of all HIV‐1 group M subtypes, and of all CRFs included in the Los Alamos National Laboratory database in 2022 (*n* = 194) [20, 21]. Phylogenetic trees were generated and genetic distances were obtained from the concatenated protease and reverse transcriptase genes by maximum likelihood methods with IQ Tree 1.6.1, using the GTR‐G nucleotide substitution model and ultrafast bootstrapping with 1000 replicates [22].

MEGA 11 software was used for tree visualization. All observations were confirmed with the available integrase genes. Transmission clusters are defined in this work as sequences (i) sharing a common ancestor, (ii) with a branch support >95% and (iii) presenting a maximum genetic distance of <4.5 substitutions per 100 nucleotides.

### 2.3 Analysis of the new CRF

We selected five strains evenly distributed on the new phylogenetic branch next to that of the CRF94 cluster, for which sufficient plasma was available for whole genome sequencing. They were amplified in the DeepChek® assay (whole‐genome HIV‐1 Genotyping, and NGS Library preparation kits, ABL, Luxembourg) with the MiSeq system (Illumina, USA). New primers were designed for the *nef* gene and the Gp120/Gp41 region (Appendix S1) for which amplification failed with the DeepChek® protocol in cases in which the HIV‐1 viral load was less than 5.5 log_10_ copies/ml.

These sequences were aligned with all the full‐length genome group M reference sequences of the Los Alamos database, and with the two CRF94 reference sequences MH141493 and MH141491. A bootscan analysis was conducted using Simplot ++ and default settings (HKY nucleotide substitution model, window length and step at 200 and 20 nucleotides, respectively) [21]. Recombination breakpoints were determined with Recombination Detection Program (RDP) version 5. Potential uncertainty intervals were determined using the 99% CI provided by RDP‐5 [23, 24]. For each fragment of single ancestry, the identified lineage was confirmed by maximum likelihood phylogenetic reconstruction with IQ Tree 1.6.1, as described above.

### 2.4 Statistical analysis

Biological parameters were compared with Mann−Whitney and Fisher's exact test for quantitative and qualitative variables, respectively. The time from treatment initiation to virological success (defined as two consecutive viral loads <50 copies/ml) was compared between the two clusters in a log‐rank test and by plotting a Kaplan−Meier curve. A multi‐variable Cox proportional hazards model was used to estimate the effect of viraemia and CD4 count at baseline, in addition to CRF and integrase inhibitor use.

### 2.5 Ethical statement and patient consent

This study was based on biological data routinely collected during patient follow‐up and did not require any additional sampling. This study did not, therefore, require ethics approval. All persons gave their written informed consent for the use of their medical chart recorded in the electronic medical record system NADIS (Fedialis Medica, France, CNIL number: 1171457, 2006), designed for the medical follow‐up of people living with HIV.

## 3 RESULTS

We analysed 98 isolates of HIV‐1 in total: 49 included in the previous CRF94 study and 49 obtained during this new collection campaign.

### 3.1 Phylogenetic analysis and characterization of the new CRF

**Figure 1:**
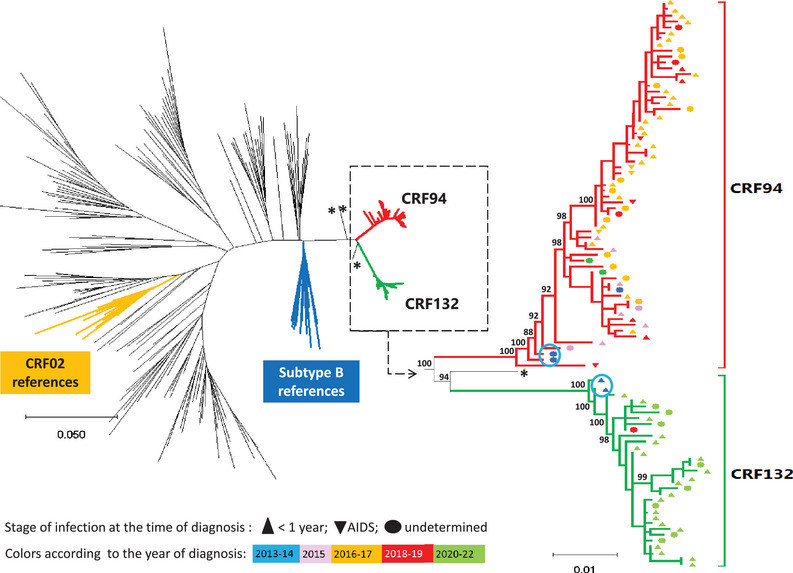
Phylogenetic trees obtained with the sequences of protease plus reverse transcriptase regions of the HIV‐1 compendium 2018 and the strains collected during the study, France, 2013–2022 (*n* = 98). The clusters CRF94 and CRF132 included 63 and 32 patients, respectively. Three were outside (*). The shortest distance between the two clusters is between infections diagnosed in 2013 (blue circle).

Phylogenetic analysis of the concatenated PR+RT sequences (1070 base pairs [bp]) and the HIV‐1 M subtype and CRF reference sequences showed that 95 of the 98 strains formed two related but separate monophyletic clades with branch support values of 100% (Figure 1). The first branch included 63 strains grouped with the CRF94 references and the second constituted a new cluster of 32 sequences different from any known reference sequence. The largest genetic distances within the CRF94 branch and the new cluster were below the maximum threshold of 4.5% and, therefore, defined transmission clusters (3.4% and 2.1%, respectively) [25]. The mean genetic distances within the clusters were below the threshold of 1.5% often used to define recent transmissions (1.36% and 0.92%, respectively). Similarly, phylogenetic analysis of the integrase gene (696 bp), which was possible for 84 of the 98 individuals, revealed the presence of two close but different branches: one corresponding to CRF94 and including 55 individuals, and the other corresponding to the new cluster with 29 persons, with maximum genetic distances of 3.8% and 1.9%, respectively (Appendix S2). Finally, three strains were located close to but outside the two clusters in the phylogenetic analyses for both the PR+RT and integrase genes (Figure 1).

**Figure 2:**
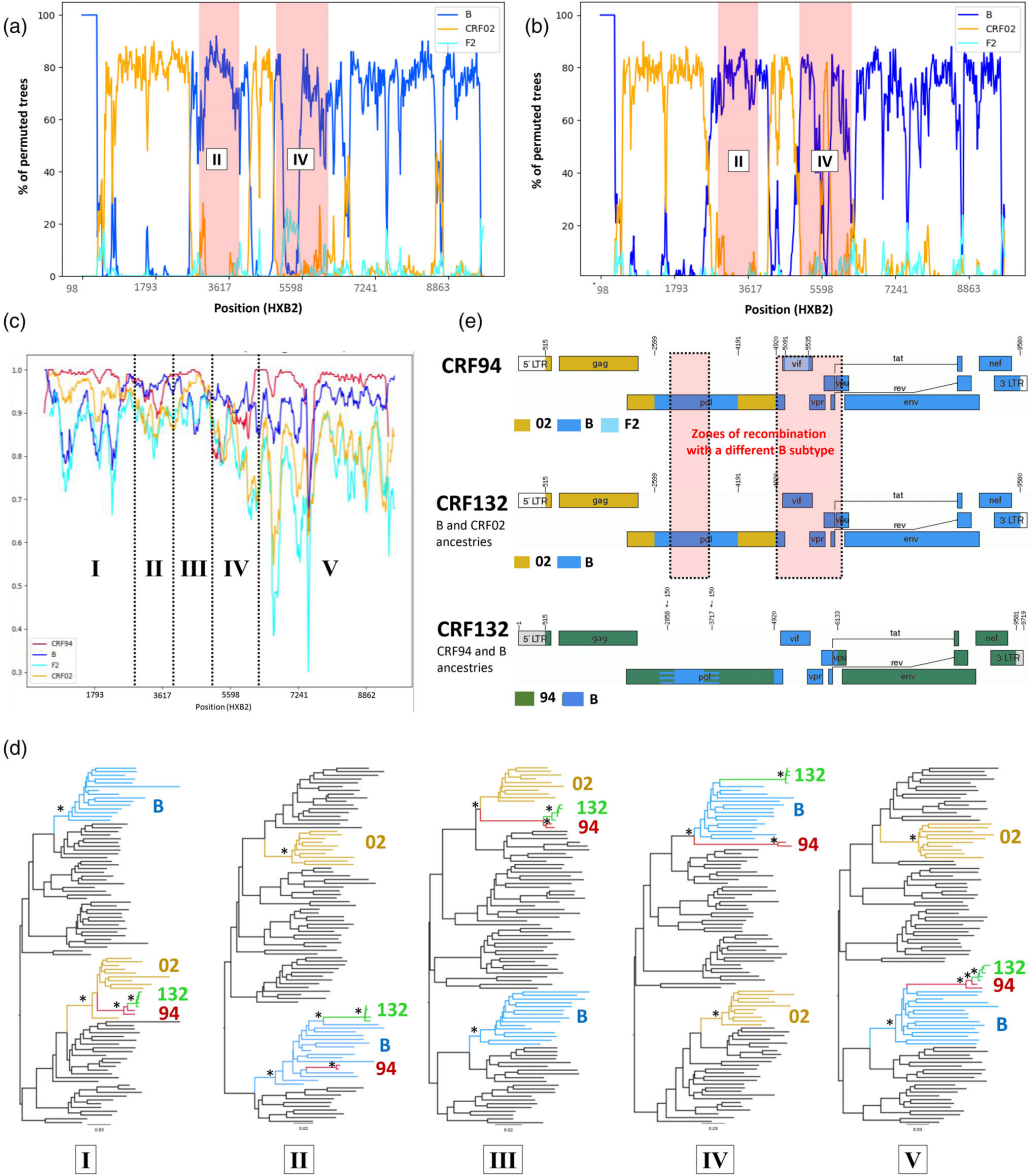
Analysis of the recombination pattern of the new cluster. The bootscan analysis of the CRF94 (Genbank reference: MH141491) and CRF132 (ON901787) against their two main ancestral lineages, that is sub‐type B (K03455—line dark blue) and CRF02 (AB485636—line yellow) are depicted in panels A and B, respectively. The red regions illustrate the region presenting significant differences between CRF94 and CRF132 as identified in the Simplot analysis of CRF132 with its potential ancestral lineages (sub‐type B, CRF02, F2 and CRF94), provided in panel C. The region IV corresponding to the accessory genes. The phylogenetic reconstruction of five areas identified with the Simplot analysis is provided in panel D, breakpoints were confirmed using RDP5. Ancestral nodes of interest with a branch support >90% are indicated by an asterisk. Panel E provides the map of recombination breakpoints identified for CRF94 and CRF132 using subtype B and CRF02 as parental lineage to highlight differences with CRF94 and for CRF132 using its direct parental lineages, CRF94 and subtype B. The alignment positions are provided using HXB2 as reference.

We characterized the strain underlying the new cluster by analysing the recombination patterns of five complete genome sequences selected as described above in the Methods section. The bootstrap results for the CRF94 references and the new cluster sequences relative to the main parental strains (i.e. subtype B and CRF02_AG) revealed slight differences in the two regions of the genome (Figure 2A,B). The Simplot analysis, comparing the variant to CRF94 and all parental lineages (subtype B, CRF02 and F2), identified five areas of the genome (Figure 2C). Breakpoints were confirmed with RDP5. For areas I, III and V of the new strain, the most similar lineage was CRF94, whereas for areas II and IV, the most similar lineage was subtype B. This suggests that the new cluster corresponds to a new lineage generated by the recombination of CRF94 with another subtype B strain within these two regions. This B strain differs from the B strain already contributing to the CRF94 sequence in this part of the genome (Figure 2E). This recombination resulted in a replacement of the F2 *vif* gene sequence (present in CRF94) with the corresponding sequence from subtype B (within the new lineage). All these observations were confirmed by phylogenetic reconstructions for each of the areas identified (Figure 2D and Appendix S3). The results of the recombination breakpoint analysis were validated by the Los Alamos National Laboratory (LANL), which registered the new lineage as CRF132_94B.

All sequences were deposited in the Genbank database (Appendix S4).

### 3.2 Epidemiological and biological comparison between the two clusters

**Figure 3:**
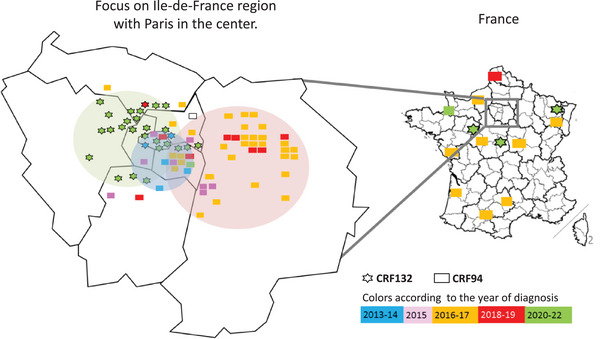
Geographic distribution of residence for the patients who were involved in two different HIV transmission cluster, France, 2013–2022 (*n* = 95). The pink and green zones show the main spreading areas for CRF94 and CRF132, respectively. The blue zone, centred south of Paris, shows the common area where the first CRF94 and CRF132 infections were detected.

The five patients diagnosed in 2013 from each cluster lived in a limited geographic area centred on the south of Paris (Figure 3). CRF94 subsequently spread massively to the east of IDF, whereas CRF132 spread to the west of IDF. Both strains eventually spread throughout the country, with 13 patients diagnosed with one of the two variants in other regions of France.

This new collection campaign identified 12 new individuals diagnosed with strains from the CRF94 cluster between 2016 and 2019, but only two persons were diagnosed since 2020. Conversely, 29 of the 32 individuals (91%) with CRF132 were diagnosed in 2021–2022. Both the CRF94 and CRF132 clusters contained at least 95% of MSM. Most of them had acquired HIV less than 12 months before diagnosis (56% and 72% for the CRF94 and CRF132 clusters, respectively), having tested negative in the preceding year (15 CRF94, 6 CRF132) or being in the acute phase (20 CRF94, 17 CRF132) at the time of diagnosis. People from the CRF94 cluster were significantly older than those from the CRF132 cluster (median age of 34 years [IQR: 28–43], vs. 30 years [IQR: 25–33], respectively, *p* = 0.018). All 63 persons included in the CRF94 cluster were of European origin, whereas 10 of the 32 (31%) individuals with CRF132 were of North African origin, although all but three were born in France. Finally, for the 47 individuals diagnosed after 2015 (when PrEP was accessible) for whom the information was available, 33% (9/27) of the persons in the CRF94 cluster were aware of PrEP versus 95% (19/20) of those in the CRF132 cluster. None of the individuals in the CRF94 cluster had used PrEP, whereas five of those diagnosed with CRF132 had previously taken PrEP but had stopped using it before diagnosis.

At the time of diagnosis, HIV‐1 viral loads were significantly higher (median: 5.42 [IQR: 4.88−5.98] vs. 4.42 [IQR: 3.78−5.33] log_10_ copies/ml, *p*<0.001), and CD4 cell counts were significantly lower (median: 358 [IQR: 199−550] vs. 508 [IQR: 414−686]/mm^3^, *p* = 0.0017) for people with CRF94 than for those with CRF132 (Table 1). These differences remained statistically significant after the exclusion of individuals in the acute phase at the time of diagnosis.

**Table 1:** Comparison of epidemiological and biological parameters between patients of CRF94 and CRF132 clusters, at the time of diagnosis

Characteristics	Patients in CRF94 cluster *n* = 63	Patients in CRF132 cluster *n* = 32	*p*‐value
**Epidemiological parameters**, *n* (%)			
Men who have sex with men	60 (95)	31 (97)	
Year of diagnosis			
2013−2019	61 (97)	3 (9)	
2020−2022	2 (3)	29 (91)	
Infection date			
Less than 1 year	35 (56)	3 (72)	
AIDS stage	6 (9)	0	0.1041
Indeterminate	18 (35)	9 (28)	
**Age and biological parameters**, median value (Interquartile range)			
Age in years	34 (28−43)	30 (25−33)	0.0183
HIV‐1 RNA (log_10_ copies/ml)			
All HIV infection stages	5.42 (4.88−5.98)	4.42 (3.78−5.33)	0.0006
Acute infections excluded	5.22 (4.81−5.74)	4.49 (3.92−5.15)	0.0100
CD4 count (cells/mm^3^)			
All HIV infection stages	358 (199−550)	508 (414−686)	0.0017
Acute infections excluded	258 (159−408)	482 (377−716)	0.0015

**Figure 4:**
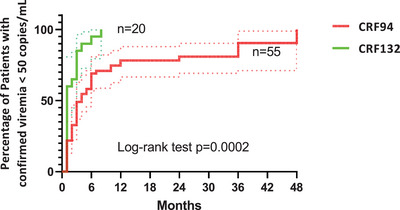
Kaplan−Meier survival curve comparing the time to achieve two consecutive viral loads <50 copies/ml after treatment initiation depending on the cluster.

Finally, it took significantly longer to achieve virological success in persons with CRF94 (hazard ratio, 2.23; 95% CI, 1.16−4.28; *p* = 0.0002) (Figure 4). The proportion of individuals treated with integrase inhibitors—a class of antiretroviral drugs known to accelerate the decrease in viraemia—did not differ significantly between the two clusters (69% vs. 75% for the CRF94 and CRF132 clusters, respectively). Multivariable Cox model analysis showed that baseline viral load was independently associated with the rapidity with which virological success was achieved (hazard ratio, 0.36 per log_10_; 95% CI, 0.25−.51; *p*<0.0001), and with CD4 count (hazard ratio per 100 CD4, 1.10; 95% CI, 1.0−1.22; *p* = 0.0027). In this model, neither the CRF clade nor the use of an integrase inhibitor had any significant effect on time to virological success.

## 4 DISCUSSION

The identification of phylogenetic clusters involving the new CRF94 strain in 2017 was followed by prevention activities in 2018 targeting the identified population and area. We show here that the number of CRF94 diagnoses decreased sharply after 2019. It is not possible to conclude that there is a cause−effect relationship from these observations for a single cluster, but our observations support the use of HIV sequence data and epidemiological information for newly diagnosed persons for the detection of HIV transmission clusters and efforts to control the spread of HIV, at least in a targeted geographic area [15]. In 2017, questionnaires including specific questions relating to the circumstances of HIV acquisition made it possible to identify the area and the population to be targeted in preventive actions. However, these retrospective investigations were laborious and time‐consuming, delaying action. Given that 5500−6300 individuals per year were found to have acquired HIV between 2013 and 2022 in France, these two clusters would not be expected to have a detectable impact on the epidemiology of HIV in France. We, therefore, recommend the development of a specific programme involving a co‐ordinated network of virologists, clinicians and actors involved in local prevention, with the support of regional or national health authorities, to expand and facilitate such actions. In the United States, the HIV prevention programme of the CDC formed the central pillar of the federal government's 2019 plan to decrease the rate of HIV acquisition in the country by 90% by 2030 [10]. The implementation of such a programme involves technical challenges in the rapid retrieval and analysis of large amounts of biological, molecular and epidemiological data, which must be overcome, with a need for strict compliance with data security and confidentiality. These constraints may partly explain the absence of such a programme in France today. As a result, we were unable to use the same resources as in 2017, which decreased the chances of identifying specific targets, and no measures could be taken to prevent the spread of the new CRF132 cluster. However, the comparison of the CRF94 and CRF132 clusters provided important information about the epidemiological and molecular factors involved in HIV dissemination.

Several of the methods used, including Simplot, RDP5 and phylogenetic reconstruction, showed that the CRF94 and CRF132 strains were very close phylogenetically. The genetic distances within and between the clusters were also very small, preventing any robust estimation of the time to the most recent common ancestor. However, the available epidemiological data revealed that these two clusters affected different populations. Consistent with our previous study, the few individuals diagnosed with CRF94 after 2017 lived in the east of the IDF region. The population acquiring CRF132 resided mostly in the west of the IDF region, was younger, and a third of these individuals were of North African origin. Finally, the CRF132 cluster expanded in 2021–2022, whereas only two patients have been diagnosed with CRF94 since 2020. This decrease in CRF94 acquisition could be due to the targeted preventive actions performed in the cluster area, or the spontaneous expansion of PrEP use after 2020. The emergence of the new cluster reveals that these assumptions do not apply to individuals with CRF132 strains. Despite being aware of PrEP, the vast majority of individuals in this cluster did not use PrEP or stopped using it before they acquired HIV. PrEP is known to be highly effective, but it is challenging to maintain compliance over time [26, 27, 28]. Recent studies reported superior efficacy for long‐acting cabotegravir or lenacapavir injections, but these regimens are not yet available for use as PrEP in France [29, 30, 31]. HIV risk perception and stigma may also act as barriers to PrEP acceptability and adherence due to the perceived association with the gay community [32, 33, 34]. Sub‐populations of MSM, such as individuals of North African origin over‐represented in the CRF132 cluster, may be particularly concerned about this stigma. Moreover, these two clusters principally concerned populations living in the suburbs rather than within Paris itself, potentially suggesting that there may be inadequate access to preventive health services and PrEP in certain areas. Further investigations are required to test these hypotheses and to identify specific populations and/or geographic areas for targeting in campaigns to increase awareness and to improve the access to and acceptance of PrEP.

Both CRF94 and CRF132 strains were present in the southern Parisian region in 2013. There is no clear evidence as to which of these two CRFs is the predecessor of the other, but the patterns of recombination observed are most consistent with a recombination of CRF94 with a subtype B strain in two parts of its genome to generate CRF132. A recombination of CRF132 with two different subtypes, B and F2, to generate CRF94 is less probable, because it would require two recombination steps rather than one. These two strains were involved in two different clusters of transmission, in the east of the IDF region in 2016–2019 for CRF94, and in the west of the region in 2021–2022 for CRF132. It was possible to identify these two new recombinant forms only when they were acquired by a large number of individuals over a short period of time. This explains the interval between the discoveries of the two strains even though both were already circulating in 2013. These results highlight the high potential of HIV to recombine over a relatively short period. The emergence of recombinants requires the acquisition of two different viral strains (here a CRF94 strain and a subtype B strain) by a single individual in the absence of antiretroviral treatment. People with multiple sex partners over a period too short for diagnosis and treatment to occur may, therefore, promote such recombination. Previous studies reported similar observations with smaller clusters. Lai et al. described the first Italian CRF (CRF60_BC) in a local outbreak in MSM between 2009 and 2011 [35]. Initially detected in Apulia, it spread among other MSM across Italy, with the outbreak also involving second‐generation recombinants originating from CRF60. In Cyprus, the CRF91_cpx and subsequent recombinants were also described in MSM [36]. These observations highlight the role of the MSM transmission clusters in the spread of HIV and new variants, and the importance of identifying these chains of transmission rapidly. A corollary is the role of late diagnosis and adherence to treatment or PrEP in the emergence and continued expansion of selected virulent strains.

The high level of viraemia observed in people with CRF94 strains may also favour recombination, in addition to transmission. Together with low CD4 cell counts, these high levels of viraemia before treatment are associated with a delayed virological response after treatment initiation. Interestingly, the most significant difference between the CRF94 and CRF132 genomes is the replacement of the subtype F2 *vif* gene with the subtype B gene. Studies in Spain have also reported higher viraemia and lower CD4 cell counts in people with subtype F than in people with subtype B [37]. This may be specific to the localized F cluster, but the authors also reported a delayed virological response to treatment in people with subtype F strains. Apolipoprotein B mRNA‐editing catalytic polypeptide (APOBEC3) cytidine deaminases are intracellular proteins that inhibit HIV‐1 replication in humans. The HIV‐1 Vif protein counteracts APOBEC3 activity by mediating the degradation of this protein by the proteasome. In a comparative study, Binka et al. observed, *in vitro*, that the highest activities against three APOBEC3 proteins were attributed to HIV strains transfected with a subtype F *vif* gene [38]. Thus, although the use of the F2 sub‐subtype was not specified and these results could be isolate‐specific, we cannot exclude the possibility that the CRF94 Vif protein contributes to the high replicative capacity of these strains. This would affect viral load and, therefore, the time required to achieve therapeutic efficacy.

## 5 CONCLUSIONS

This study shows that the surveillance of HIV transmission clusters with sequencing data from genotypic resistance testing can provide important information about several aspects facilitating containment of the HIV epidemic. The first is the possibility of identifying a geographic area and a specific population involved in a chain of transmission and targeting them with preventive actions to stop the spread. Surveillance can also be used to assess the efficacy of such interventions. The major decreases in the number of diagnoses of CRF94 acquisition after 2018 may be attributed to such actions, at least in part. However, resources and an appropriate organization are required to maximize the chances of success. The absence of an appropriate programme makes it difficult to detect these chains of transmission rapidly and to implement targeted preventive actions promptly. Such programmes can also help to identify sub‐populations at risk but reluctant to use PrEP due to sigma or inequities in PrEP access. It is, thus, possible to implement appropriate actions to improve the acceptance of and access, and adherence to this key strategy as a means of curtailing the HIV epidemic. They would also make it possible to detect the emergence of potentially more virulent HIV variants, the spread of which could be halted through surveillance measures. Finally, molecular studies of such strains can also shed light on virulence mechanisms, as illustrated by the possible role of the *vif* gene in determining viral load level for HIV.

## COMPETING INTERESTS

The authors have no competing interests.

## AUTHORS’ CONTRIBUTIONS

MW, FDO, MB‐A, J‐CP, AG‐M and BV were involved in the conceptualization of the research question and coordinated the data collection. MW, FDO and BV contributed with molecular analysis, analysed the data and wrote the manuscript. MB‐A, MBV, SL‐N, ML‐C, SR, ASM, CA, EAG, PB, BM, AM, CP, JDP, TR, VS, ASS, KS, VC and DD contributed to epidemiological data collection from respective regions. All authors reviewed and approved the final version of the manuscript.

## FUNDING

This work was supported by ANRS‐MIE (Agence Nationale de Recherche sur le SIDA et les Hépatites Virales‐Maladies Infectieuses Emergentes).

## PRIOR PRESENTATIONS

This work has been presented in part at the CROI 2023 conference: Abstract #844.

## Supporting information



Appendix S1

Appendix S2

Appendix S3

Appendix S4

## Data Availability

The following sequences were deposited in the GenBank database: the five near‐complete genomes of CRF132 (ON901787‐ON901791); 30 PT‐RT sequences (PQ554426‐PQ554455) including the other CRF132 strains and the three non‐typable strains (PQ554427‐PQ554428‐PQ554426); 57 PT‐RT sequences from CRF94 (PQ554456‐PQ554512). The six near‐complete genome sequences for CRF94 had already been deposited: accession numbers MH141491 to MH141494 and MH683549, MH683550.

## References

[1] HIV—Global [Internet]. [cited 2024 Oct 25]. Available from: https://www.who.int/health‐topics/hiv‐aids

[2] Haar K , Amato‐Gauci AJ . European men who have sex with men still at risk of HIV infection despite three decades of prevention efforts. Euro Surveill. 2015;20(14):21087. 25884146 10.2807/1560-7917.es2015.20.14.21087

[3] Stengaard AR , Combs L , Supervie V , Croxford S , Desai S , Sullivan AK , et al. HIV seroprevalence in five key populations in Europe: a systematic literature review, 2009 to 2019. Euro Surveill. 2021;26(47):2100044. 34823636 10.2807/1560-7917.ES.2021.26.47.2100044 PMC8619876

[4] Luo S , Han L , Lu H , Dou Z , Tao Q , Khoshnood K , et al. Evaluating the impact of test‐and‐treat on the HIV epidemic among MSM in China using a mathematical model. PLoS One. 2015;10(6):e0126893. 26039075 10.1371/journal.pone.0126893 PMC4454496

[5] Havlir D , Lockman S , Ayles H , Larmarange J , Chamie G , Gaolathe T , et al. What do the Universal Test and Treat trials tell us about the path to HIV epidemic control? J Int AIDS Soc. 2020;23(2):e25455. 32091179 10.1002/jia2.25455 PMC7038879

[6] Brizzi F , Birrell PJ , Kirwan P , Ogaz D , Brown AE , Delpech VC , et al. Tracking elimination of HIV transmission in men who have sex with men in England: a modelling study. Lancet HIV. 2021;8(7):e440–e448. 34118196 10.1016/S2352-3018(21)00044-8 PMC8238681

[7] Erly SJ , Herbeck JT , Kerani RP , Reuer JR . Characterization of molecular cluster detection and evaluation of cluster investigation criteria using machine learning methods and statewide surveillance data in Washington state. Viruses. 2020;12(2):142. 31991877 10.3390/v12020142 PMC7077225

[8] Oster AM , France AM , Mermin J . Molecular epidemiology and the transformation of HIV prevention. JAMA. 2018;319(16):1657–1658. 29630701 10.1001/jama.2018.1513 PMC12599837

[9] Oster AM , France AM , Panneer N , Bañez Ocfemia MC , Campbell E , Dasgupta S , et al. Identifying clusters of recent and rapid HIV transmission through analysis of molecular surveillance data. J Acquire Immune Defic Syndr. 2018;79(5):543. 10.1097/QAI.0000000000001856 PMC6231979 30222659

[10] Oster AM , Lyss SB , McClung RP , Watson M , Panneer N , Hernandez AL , et al. HIV cluster and outbreak detection and response: the science and experience. Am J Prev Med. 2021;61(5):S130–S142. 34686282 10.1016/j.amepre.2021.05.029 PMC12599833

[11] Visseaux B , Assoumou L , Mahjoub N , Grude M , Trabaud MA , Raymond S , et al. Surveillance of HIV‐1 primary infections in France from 2014 to 2016: toward stable resistance, but higher diversity, clustering and virulence? J Antimicrob Chemother. 2020;75:183–193. 31641777 10.1093/jac/dkz404

[12] Poon AFY , Gustafson R , Daly P , Zerr L , Demlow SE , Wong J , et al. Near real‐time monitoring of HIV transmission hotspots from routine HIV genotyping: an implementation case study. Lancet HIV. 2016;3(5):e231–e238. 27126490 10.1016/S2352-3018(16)00046-1 PMC4853759

[13] Chaillon A , Essat A , Frange P , Smith DM , Delaugerre C , Barin F , et al. Spatiotemporal dynamics of HIV‐1 transmission in France (1999–2014) and impact of targeted prevention strategies. Retrovirology. 2017;14:15. 28222757 10.1186/s12977-017-0339-4 PMC5322782

[14] González‐Domenech CM , Viciana I , Delaye L , Mayorga ML , Palacios R , de la Torre J , et al. Emergence as an outbreak of the HIV‐1 CRF19_cpx variant in treatment‐naïve patients in southern Spain. PLoS One. 2018;13(1):e0190544. 29309418 10.1371/journal.pone.0190544 PMC5757947

[15] Wirden M , De Oliveira F , Bouvier‐Alias M , Lambert‐Niclot S , Chaix ML , Raymond S , et al. New HIV‐1 circulating recombinant form 94: from phylogenetic detection of a large transmission cluster to prevention in the age of geosocial‐networking apps in France, 2013 to 2017. Euro Surveill. 2019;24(39):1800658. 31576801 10.2807/1560-7917.ES.2019.24.39.1800658 PMC6774227

[16] Wymant C , Bezemer D , Blanquart F , Ferretti L , Gall A , Hall M , et al. A highly virulent variant of HIV‐1 circulating in the Netherlands. Science. 2022;375(6580):540–545. 35113714 10.1126/science.abk1688

[17] Williams A , Menon S , Crowe M , Agarwal N , Biccler J , Bbosa N , et al. Geographic and population distributions of human immunodeficiency virus (HIV)–1 and HIV‐2 circulating subtypes: a systematic literature review and meta‐analysis (2010–2021). J Infect Dis. 2023;228(11):1583–1591. 37592824 10.1093/infdis/jiad327 PMC10681860

[18] EACSociety . EACS Guidelines [Internet]. [cited 2024 Oct 25]. Available from: https://www.eacsociety.org/guidelines/eacs‐guidelines/

[19] CNS W . Recommandations de prise en charge du VIH, des hépatites virales et des IST : rapport d'experts [Internet]. Conseil national du sida et des hépatites virales. 2023 [cited 2024 Oct 25]. Available from: https://cns.sante.fr/dossiers/dossier‐experts/rapport‐experts‐2023/

[20] Larkin MA , Blackshields G , Brown NP , Chenna R , McGettigan PA , McWilliam H , et al. Clustal W and Clustal X version 2.0. Bioinformatics. 2007;23(21):2947–2948. 17846036 10.1093/bioinformatics/btm404

[21] Tamura K , Stecher G , Kumar S . MEGA11: molecular evolutionary genetics analysis version 11. Mol Biol Evol. 2021;38(7):3022–3027. 33892491 10.1093/molbev/msab120 PMC8233496

[22] Minh BQ , Schmidt HA , Chernomor O , Schrempf D , Woodhams MD , von Haeseler A , et al. IQ‐TREE 2: new models and efficient methods for phylogenetic inference in the genomic era. Mol Biol Evol. 2020;37(5):1530–1534. 32011700 10.1093/molbev/msaa015 PMC7182206

[23] Martin DP , Varsani A , Roumagnac P , Botha G , Maslamoney S , Schwab T , et al. RDP5: a computer program for analyzing recombination in, and removing signals of recombination from, nucleotide sequence datasets. Virus Evol. 2021;7(1):veaa087. 33936774 10.1093/ve/veaa087 PMC8062008

[24] Samson S , Lord É , Makarenkov V . SimPlot++: a Python application for representing sequence similarity and detecting recombination. Bioinformatics. 2022;38(11):3118–3120. 35451456 10.1093/bioinformatics/btac287

[25] Hassan AS , Pybus OG , Sanders EJ , Albert J , Esbjornsson J . Defining HIV‐1 transmission clusters based on sequence data. AIDS. 2017;31(9):1211–1222. 28353537 10.1097/QAD.0000000000001470 PMC5482559

[26] Grant RM , Lama JR , Anderson PL , McMahan V , Liu AY , Vargas L , et al. Preexposure chemoprophylaxis for HIV prevention in men who have sex with men. N Engl J Med. 2010;363(27):2587–2599. 21091279 10.1056/NEJMoa1011205 PMC3079639

[27] McCormack S , Dunn DT , Desai M , Dolling DI , Gafos M , Gilson R , et al. Pre‐exposure prophylaxis to prevent the acquisition of HIV‐1 infection (PROUD): effectiveness results from the pilot phase of a pragmatic open‐label randomised trial. Lancet. 2016;387(10013):53–60. 26364263 10.1016/S0140-6736(15)00056-2 PMC4700047

[28] Molina JM , Ghosn J , Assoumou L , Delaugerre C , Algarte‐Genin M , Pialoux G , et al. Daily and on‐demand HIV pre‐exposure prophylaxis with emtricitabine and tenofovir disoproxil (ANRS PREVENIR): a prospective observational cohort study. Lancet HIV. 2022;9(8):e554–e562. 35772417 10.1016/S2352-3018(22)00133-3

[29] Delany‐Moretlwe S , Hughes JP , Bock P , Ouma SG , Hunidzarira P , Kalonji D , et al. Cabotegravir for the prevention of HIV‐1 in women: results from HPTN 084, a phase 3, randomised clinical trial. Lancet. 2022;399(10337):1779–1789. 35378077 10.1016/S0140-6736(22)00538-4 PMC9077443

[30] Kelley CF , Acevedo‐Quiñones M , Agwu AL , Avihingsanon A , Benson P , Blumenthal J , et al. Twice‐yearly lenacapavir for HIV prevention in men and gender‐diverse persons. N Engl J Med. 2024 10.1056/NEJMoa2411858 39602624

[31] Bekker LG , Das M , Abdool Karim Q , Ahmed K , Batting J , Brumskine W , et al. Twice‐yearly lenacapavir or daily F/TAF for HIV prevention in cisgender women. N Engl J Med. 2024;391(13):1179–1192. 39046157 10.1056/NEJMoa2407001

[32] Kamitani E , Wichser ME , Mizuno Y , DeLuca JB , Higa DH . What factors are associated with willingness to use HIV pre‐exposure prophylaxis (PrEP) among U.S. men who have sex with men not on PrEP?: a systematic review and meta‐analysis. J Assoc Nurses AIDS Care. 2023;34(2):135–145. 36563302 10.1097/JNC.0000000000000384 PMC10184317

[33] Sidebottom D , Ekström AM , Strömdahl S . A systematic review of adherence to oral pre‐exposure prophylaxis for HIV—how can we improve uptake and adherence? BMC Infect Dis. 2018;18(1):581. 30445925 10.1186/s12879-018-3463-4 PMC6240194

[34] Chou R , Evans C , Hoverman A , Sun C , Dana T , Bougatsos C , et al. Preexposure prophylaxis for the prevention of HIV infection: evidence report and systematic review for the US Preventive Services Task Force. JAMA. 2019;321(22):2214–2230. 31184746 10.1001/jama.2019.2591

[35] Lai A , Simonetti FR , Brindicci G , Bergna A , Di Giambenedetto S , Sterrantino G , et al. Local epidemics gone viral: evolution and diffusion of the Italian HIV‐1 recombinant form CRF60_BC. Front Microbiol. 2019;10:769. 31031735 10.3389/fmicb.2019.00769 PMC6474184

[36] Topcu C , Georgiou V , Rodosthenous JH , Demetriades I , Foley BT , G Kostrikis L . Characterization of a novel HIV‐1 circulating recombinant form, CRF91_cpx, comprising CRF02_AG, G, J, and U, mostly among men who have sex with men. Virulence. 2022;13(1):1331–1348. 35979885 10.1080/21505594.2022.2106021 PMC9397478

[37] Pernas B , Grandal M , Mena A , Castro‐Iglesias A , Cañizares A , Wyles DL , et al. High prevalence of subtype F in newly diagnosed HIV‐1 persons in northwest Spain and evidence for impaired treatment response. AIDS. 2014;28(12):1837–1840. 24871456 10.1097/QAD.0000000000000326

[38] Binka M , Ooms M , Steward M , Simon V . The activity spectrum of Vif from multiple HIV‐1 subtypes against APOBEC3G, APOBEC3F, and APOBEC3H. J Virol. 2012;86(1):49–59. 22013041 10.1128/JVI.06082-11 PMC3255910

