# Supplementary material for: Benefits of HIV‐1 transmission cluster surveillance: a French retrospective observational study of the molecular and epidemiological co‐evolution of recent circulating recombinant forms 94 and 132
Source: J Int AIDS Soc. 2025 Jan 28;28(2):e26416. doi: 10.1002/jia2.26416 (PMC11774651; doi:10.1002/jia2.26416)
Supplement: Supplementary file 1 — Appendix S1 [file JIA2-28-e26416-s003.docx]

Appendix S1 :

New primers were designed for the *nef* gene and the Gp120/Gp41 region for which amplification failed with the DeepChek® protocol in cases in which the HIV-1 viral load was less than 5.5 log_10_ copies/mL :

To amplify the Gp120/Gp41 region (HXB2 region : 5970-8022) :

|  |  | Primer (5’-3’) |
| --- | --- | --- |
| PCR | Outer Primer 1 | TAGGCATYTCCTATGGCAGGAAGAA |
|  | Outer Primer 2 | GTGAGTATCCCTGCCTAACTCTAT |
| nested PCR | Inner Primer 1 | ATGGCAGGAAGAAGCGGARRC |
|  | Inner Primer 2 | AGTGGTGCAAATGAGTTTTCC |

To amplify the Nef region (7798-9515) :

|  |  | Primer (5’-3’) |
| --- | --- | --- |
| PCR | Outer Primer 1 | TAGGAGCAGCAGGAAGCACTATG |
|  | Outer Primer 2 | GCAAGCTTTATTGAGSSTTAAGCAG |
| nested PCR | Inner Primer 1 | CAGCAGGWAGCACTATGGG |
|  | Inner Primer 2 | TTATATGCAGCWTCTGAGGG |
