# Supplementary material for: Benefits of HIV‐1 transmission cluster surveillance: a French retrospective observational study of the molecular and epidemiological co‐evolution of recent circulating recombinant forms 94 and 132
Source: J Int AIDS Soc. 2025 Jan 28;28(2):e26416. doi: 10.1002/jia2.26416 (PMC11774651; doi:10.1002/jia2.26416)
Supplement: Supplementary file 2 — Appendix S2 [file JIA2-28-e26416-s004.pptx]

## Slide 1
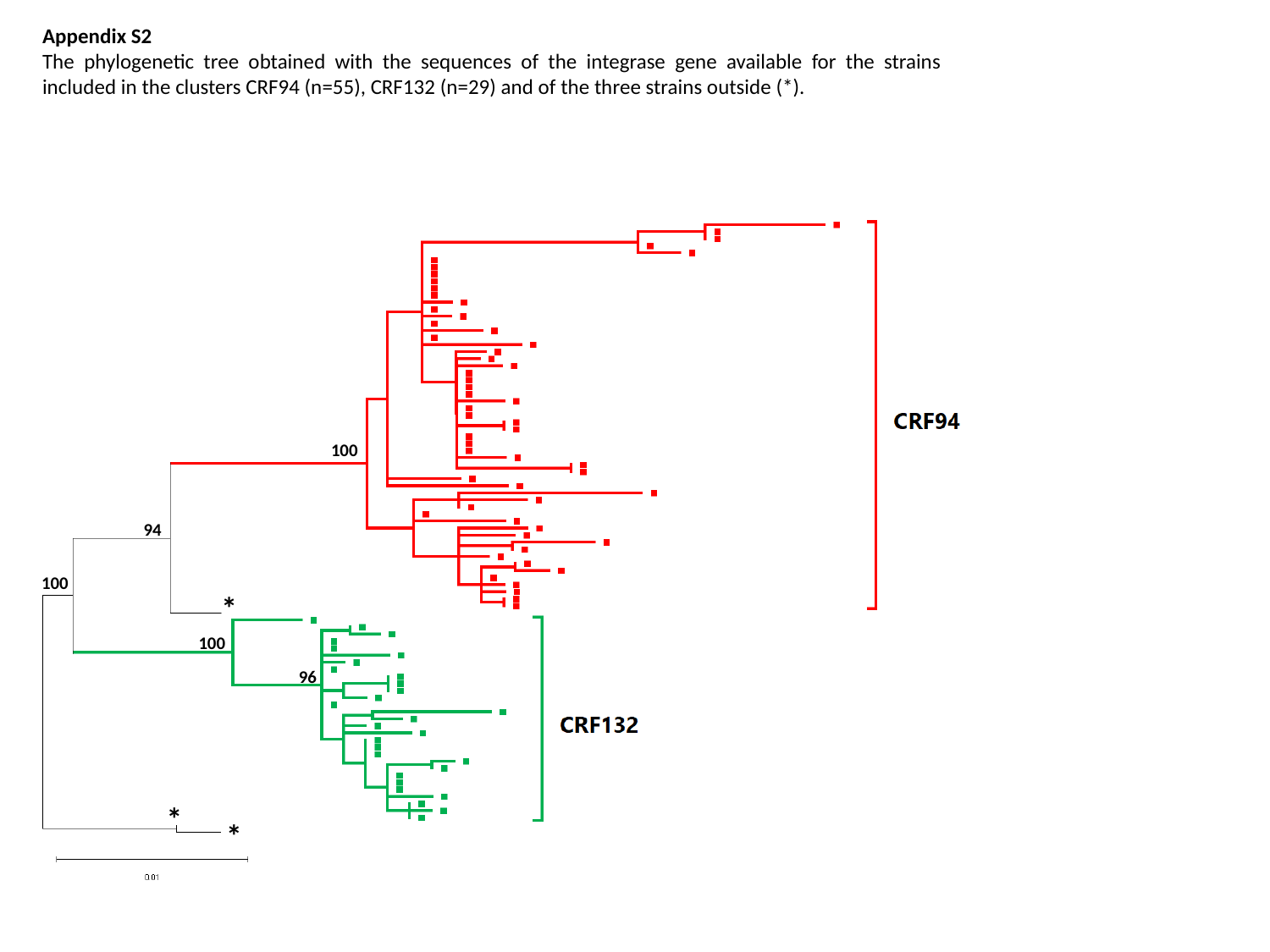

Appendix S2
The phylogenetic tree obtained with the sequences of the integrase gene available for the strains included in the clusters CRF94 (n=55), CRF132 (n=29) and of the three strains outside (*).
100
94
100
*
100
96
*
*
