# Supplementary material for: Benefits of HIV‐1 transmission cluster surveillance: a French retrospective observational study of the molecular and epidemiological co‐evolution of recent circulating recombinant forms 94 and 132
Source: J Int AIDS Soc. 2025 Jan 28;28(2):e26416. doi: 10.1002/jia2.26416 (PMC11774651; doi:10.1002/jia2.26416)
Supplement: Supplementary file 3 — Appendix S3 [file JIA2-28-e26416-s002.pptx]

## Slide 1
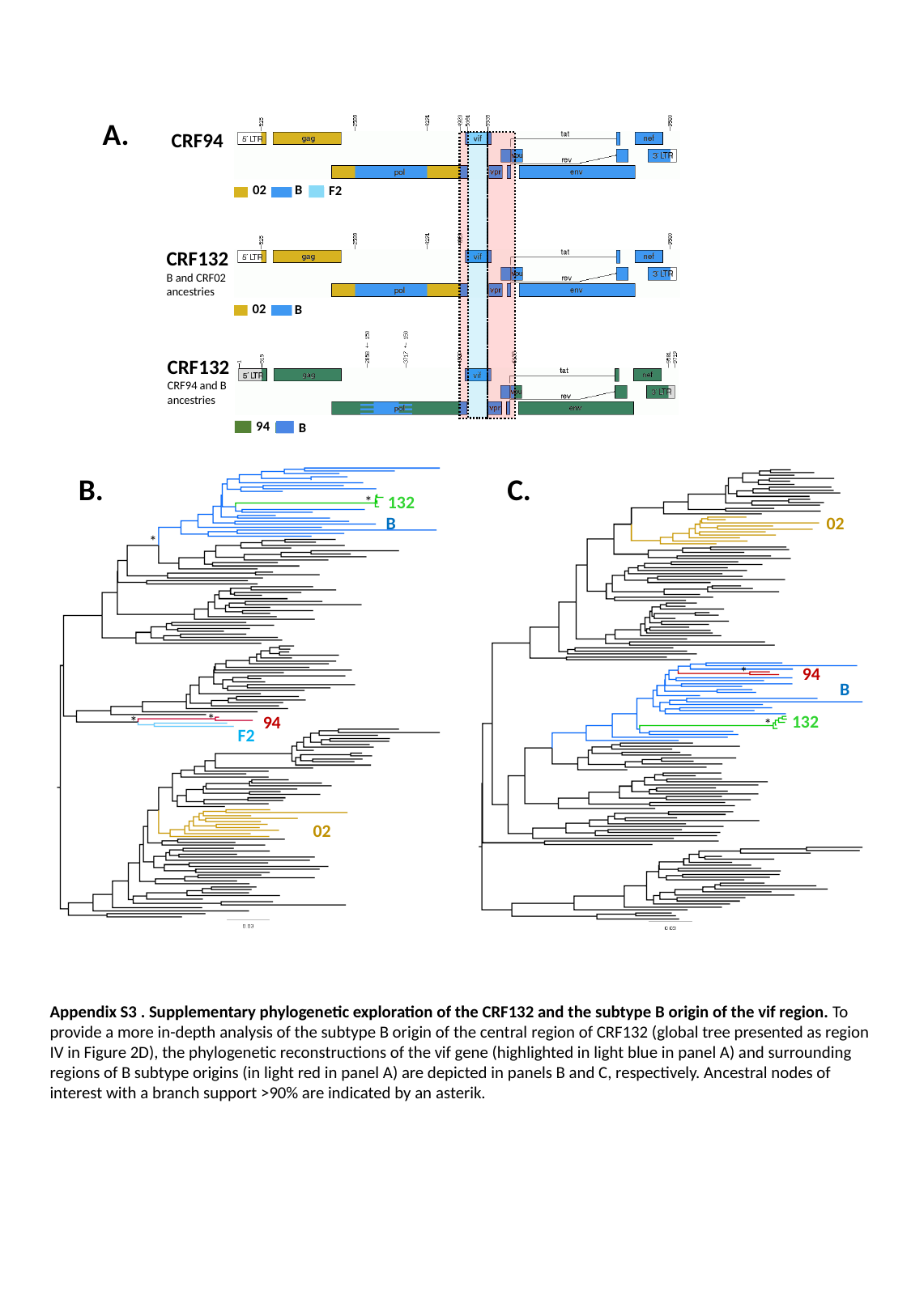

A.
CRF94
02
B
F2
CRF132
B and CRF02
ancestries
02
B
CRF132
CRF94 and B ancestries
94
B
B.
C.
132
*
B
*
*
94
*
F2
02
02
94
*
B
132
*
Appendix S3 . Supplementary phylogenetic exploration of the CRF132 and the subtype B origin of the vif region. To provide a more in-depth analysis of the subtype B origin of the central region of CRF132 (global tree presented as region IV in Figure 2D), the phylogenetic reconstructions of the vif gene (highlighted in light blue in panel A) and surrounding regions of B subtype origins (in light red in panel A) are depicted in panels B and C, respectively. Ancestral nodes of interest with a branch support >90% are indicated by an asterik.
