# Supplementary material for: Benefits of HIV‐1 transmission cluster surveillance: a French retrospective observational study of the molecular and epidemiological co‐evolution of recent circulating recombinant forms 94 and 132
Source: J Int AIDS Soc. 2025 Jan 28;28(2):e26416. doi: 10.1002/jia2.26416 (PMC11774651; doi:10.1002/jia2.26416)
Supplement: Supplementary file 4 — Appendix S4 [file JIA2-28-e26416-s001.docx]

Appendix S4

The following sequences were deposited in the GenBank database: the five near-complete genomes of CRF132 (ON901787 -ON901791); 30 PT-RT sequences (PQ554426-PQ554455) including the other CRF132 strains and the three non-typable strains (PQ554427- PQ554428- PQ554426); 57 PT-RT sequences from CRF94 (PQ554456-PQ554512). The six near-complete genomes sequences for CRF94 had already been deposited: accession numbers MH141491 to MH141494 and MH683549, MH683550
